# Apricot Seed Shells and Walnut Shells as Unconventional Sugars and Lignin Sources

**DOI:** 10.3390/molecules28031455

**Published:** 2023-02-02

**Authors:** Vita Halysh, Juan Miguel Romero-García, Alfonso M. Vidal, Tetiana Kulik, Borys Palianytsia, Minerva García, Eulogio Castro

**Affiliations:** 1 Department of Ecology and Technology of Plant Polymers, Faculty of Chemical Engineering, Igor Sikorsky Kyiv Polytechnic Institute, Peremogy Avenu 37/4, 03056 Kyiv, Ukraine; 2 Laboratory of Kinetics and Mechanisms of Chemical Reactions on the Surface of Solids, Chuiko Institute of Surface Chemistry, National Academy of Sciences of Ukraine, General Naumov Str., 17, 03164 Kyiv, Ukraine; 3 Department of Chemical, Environmental and Materials Engineering, Universidad de Jaén, Campus Las Lagunillas s/n, 23071 Jaén, Spain; 4 Center for Advanced Studies in Earth Sciences, Energy and Environment (CEACTEMA), Universidad de Jaén, Campus Las Lagunillas s/n, 23071 Jaén, Spain; 5 Tecnológico Nacional de México/Instituto Tecnológico de Zitácuaro, Av. Tecnológico No. 186 Manzanillos, Zitácuaro 61534, Michoacán, Mexico

**Keywords:** biorefinery, enzymatic hydrolysis, lignin, pretreatment, sugars

## Abstract

The present study focuses on using apricot seeds shells and walnut shells as a potential renewable material for biorefinery in Ukraine. The goal of the research work was to determine the relationship between the chemical composition of solid residues from biomass after acid pretreatment with H_2_SO_4_, alkaline pretreatment with NaOH, and a steam explosion pretreatment and the recovery of sugars and lignin after further enzymatic hydrolysis with the application of an industrial cellulase Cellic CTec2. Apricot seeds shells and walnut shells consist of lots of cellulose (35.01 and 24.19%, respectively), lignin (44.55% and 44.63%, respectively), hemicelluloses (10.77% and 26.68%, respectively), and extractives (9.97% and 11.41%, respectively), which affect the efficiency of the bioconversion of polysaccharides to sugars. The alkaline pretreatment was found to be more efficient in terms of glucose yield in comparison with that of acid and steam explosion, and the maximum enzymatic conversions of cellulose reached were 99.7% and 94.6% for the solids from the apricot seeds shells and the walnut shells, respectively. The maximum amount of lignin (82%) in the residual solid was obtained during the processing of apricot seed shells submitted to the acid pretreatment. The amount of lignin in the solids interferes with the efficiency of enzymatic hydrolysis. The results pave the way for the efficient and perspective utilization of shells through the use of inexpensive, simple and affordable chemical technologies, obtaining value-added products, and thus, reducing the amount of environmental pollution (compared to the usual disposal practice of direct burning) and energy and material external dependency (by taking advantage of these renewable, low-cost materials).

## 1. Introduction

Global population growth is associated with an increasing demand for energy resources. In this regard, the use of oil is growing every year [1]. As a fossil energy source, the use of oil presents both environmental and availability concerns, making the search for unconventional alternative energy sources necessary. Renewable, lignocellulosic materials such as forest, agricultural, and agroindustrial residues constitute prominent examples of those potential energy sources. Contrary to fossil energy sources, renewable sources are spread all over the world, and their use does not increase the net greenhouse gases emissions.

In addition to energy, the chemical composition of these materials can be used, through different fractionation and conversion schemes, to obtain a wide range of bio-based products that can be substituted for their similar ones currently derived from fossil sources. Biofuels such as bio-oil, biochar, non-condensable gases, and bioethanol can be obtained from different types of biomasses via thermal, thermocatalytic, and biological conversion [2]. Ethanol is regarded as the most attractive and clean fuel, and it can be used in combustion engines [3]. First- and second-generation biofuels are obtained mainly from renewable plant materials. Food crops, e.g., wheat [4], corn [5], sugarcane [6], etc., are the main materials for first-generation biofuels, while lignocellulosic materials, e.g., inexpensive wastes of food and non-food industries, can be used for the biochemical processing into second-generation biofuels [7,8]. The growing interest in the development and application of new goods and materials, chemicals of an aromatic nature, and energy and fuels from renewable vegetable wastes can be observed worldwide [9,10,11] due to their widespread availability and low cost.

Different technologies, including hydrolysis, extraction, pyrolysis, and chemical modifications have been recently reviewed to summarize the progress on the production of value-added products such as polymers, bioactive compounds, and bioplastics, among others, starting with biomass and waste feedstocks [12].

Another way of converting lignocellulosic materials into useful products is the biochemical rout, which includes processing via a pretreatment, the enzymatic hydrolysis of the resulting solid, and the fermentation of hydrolysates. This procedure allows us to converse the polysaccharide component of the lignocellulose into biofuels and to obtain lignin as a residue [13]. In spite of the recent acquisition of knowledge about the bioconversion processes, it is still necessary to study the processes of biochemical conversion for individual plant materials because much of it is still not fully understood. For instance, the resistance of carbohydrates to degradation by enzymes (the so-called recalcitrance effect), which is closely related to the presence of extractives of a different nature and lignin [14,15], the amount of which in the biomass depends on its type and the growing and harvesting conditions, as well as many other factors. Pretreatment is an important stage before biological conversion, which allows it to partially overcome biomass recalcitrance. The main task of any pretreatment of biomass is the mitigation of the recalcitrance and increase in the accessibility of cellulose [16]. Even though many researchers have worked on developing different methods, including a mechanical one [17], extraction with organic solvents [18], a hydrothermal one [19], a dilute acid one [20], and an alkaline one [21], most of these papers are related to the study of the sugar yield without considering the structural changes of each biomass constituent, including lignin.

The most typical methods for raw materials pretreatment before enzymatic hydrolysis are an acid pretreatment with the application of diluted sulfuric acid at a concentration of 1%, an alkaline pretreatment with the application of 15% sodium hydroxide solution, and also, a steam explosion pretreatment [22,23,24].

The great advantage of lignocellulosic waste, including agricultural waste, is its wide distribution throughout the earth, rather than the specific locations of fossil energy sources such as oil. This means that the number of raw materials that can be used is very large. Among the different agricultural residues, those derived from the cultivation of apricots and walnuts, specifically the shells, have been studied only in a limited way up to now. Ukraine is an agro-industrial country, which annually produces a huge amount of plant wastes from the agricultural and forestry industries. According to FAO [25], during the period 2017–2021, the average production in Ukraine of apricot seed shells was 81,666 tons, and that of walnut shells was 111,088 tons, representing 8.32 and 32.74% of the European production total, as well as a disposal problem. That is why these materials were chosen as raw materials for the experimental work. Agricultural residues and forestry wastes are a lignocellulose complex [26,27] which does not have further industrial applications. Most of these residues are burned, which causes significant damage to the environment. However, their sugar composition indicates great potential for chemical processing. It is promising, from an economic point of view, to develop efficient methods to utilize them and obtain valuable materials, chemicals, and fuels [28,29].

As it is known, the lignocellulosic biomass is composed of cellulose, hemicelluloses, and lignin in different proportions. Lignin is composed of a heterogeneous polymer of phenyl-propane, and it has a recalcitrant and complex nature due to it having strong bonds. The effect of lignin on the release of sugars during the enzymatic hydrolysis of pretreated biomass has been widely studied especially because of the inhibition of enzyme action; in general, two strategies to separate the lignin fraction can be performed, depending on the pretreatment applied. On the one side, lignin can be obtained as the first fraction following an alkaline pretreatment [30] or it can constitute the solid residue after the fractionation of hemicellulose and conversion of cellulose into glucose. A delignification of biomass can improve the saccharification yield [31].

The aim of the of present research work was to assess the impact of the acid, alkaline, and steam explosion pretreatments of apricot seeds shells and walnut shells on the efficiency of enzymatic hydrolysis with an industrial cellulase Cellic CTec2 enzyme complex for the recovery of sugars and lignin, which are products used by a biorefinery in Ukraine.

## 2. Results and Discussion

### 2.1. Raw Material Composition

The chemical compositions of the initial apricot seed shells and walnut shells are given in Table 1. The analysis of the apricot seed shells composition showed that they contain less extractives compared to those in walnut shells. The amount of cellulose in the apricot seed shells is higher than it is in walnut shells, but the walnut shells are composed of a higher proportion of hemicelluloses. The amount of cellulose in the apricot seed shells agrees with data presented by other scientists, who gave a value of 34.31% [32]. Most of the glucoses are related to cellulose. The amount of xylose is the highest among all of the low-molecular-weight polysaccharides, and it is 9.41% and 24.13% for apricot seed shells and walnut shells, respectively. Both of the plant materials are composed of a high percentage of lignin (44.5 and 44.6% dry weight). Acid-insoluble lignin makes up 44.4 and 44.5% of the total lignin, with the rest being acid-soluble lignin. The amount of ash in the studied materials is lower than 0.5%. The amount of acetyl groups in walnut shells is higher than that of apricot seed shells.

Compared to the data provided by Queirós et al. [33], the amount of the sugars in the walnut shells are quite similar, while the other components are a little bit different. Compared to the chemical composition of walnut shells reported by other scientists [34], the difference in the amount of the main components is significant. Such differences can be related to the differences in the plant variety, growth conditions, climate, soils, and other factors.

### 2.2. Effect of Pretreatment on Solid Recovery

Hemicelluloses contribute to biomass recalcitrance as they cover and protect cellulose fibrils from enzymatic destruction [35]. An acid pretreatment of biomass with diluted sulfuric acid is an efficient means to solubilize low-molecular-weight polysaccharides. At the same time, an alkaline pretreatment is one of the most attractive techniques for the biomass’ preparation for further enzymatic hydrolysis because of its high selectivity for lignin separation [36]. The steam explosion pretreatment is regarded as an alternative and environmentally friendly technique that can be used to reduce biomass recalcitrance, resulting in desirable cellulose accessibility [37]. It happens due to the fact that saturated steam heats the lignocellulosic biomass and releases pressure, causing the cleavage of fibers. Additionally, the removal of extractives and the partial solubilization of hemicellulosic constituents is observed.

**Figure 1:**
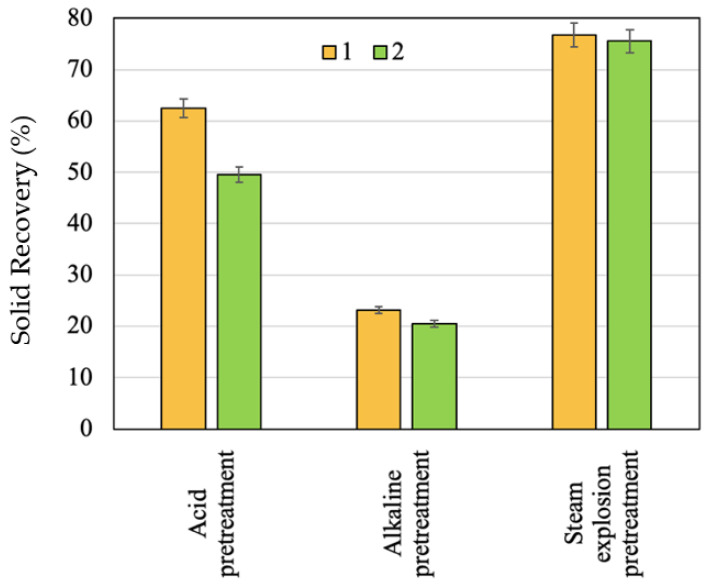
Effect of pretreatments on solid recovery: 1—apricot seed shells; 2—walnut shells.

**Figure 2:**
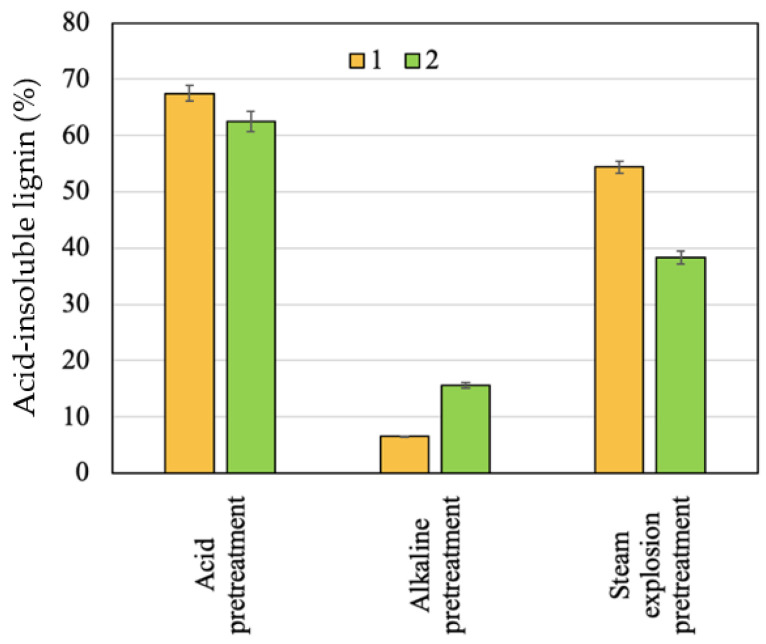
Effect of pretreatments on residual lignin content: 1—apricot seed shells; 2—walnut shells.

The effect of the raw materials pretreatments with 1% H_2_SO_4_ at 180 °C in an agitated tank reactor and with 15% NaOH at 180 °C in agitated tank reactor and with the application of steam explosion for solid recovery is shown in Figure 1 and Figure 2. Table 2 shows the results of the acid, alkali, and steam explosion (180 °C, 10 min) pretreatments in terms of solid characterization, e.g., sugars contents.

The pretreatment of apricot seed shells and walnut shells with 1% H_2_SO_4_ at 180 °C in an agitated tank reactor caused the removal of 37.54% and 50.35% by weight from raw material, respectively, and resulted in the formation of solids with more lignin, while the amount of cellulose is still high (based on glucose content, it is greater than 30%), but it is slightly lower than it was in the initial material. Lignin and cellulose were less solubilized, thus both of the components are the major constituents in the solid. Similar results in terms of solid recovery were obtained by Benjamin et al. [38] during a bagasse sugarcane pretreatment with diluted acid: the solid recovery ranged from 50.1% to 76.5% depending on the parameters of the process. A high solid recovery value after the acid pretreatment was obtained for the apricot seed shells. This may be due to there being more cellulose in the initial biomass.

The loss of 76.79% and 79.5% of the mass during the alkali pretreatment of the apricot seed shells and walnut shells with 15% NaOH at 180 °C in an agitated tank reactor, respectively, is related to delignification. The amount of lignin in the solids was 6.7 and 2.8 times lower in comparison with those of the initial apricot seed shells and walnut shells, respectively. Under the same conditions, the apricot seed shells were more easily delignified than the walnut shells were. The corn stover alkaline pretreatment resulted in the dissolution of 50% of the hemicellulose and of 60–80% of the lignin [39]. The reduction in lignin content to 9.50% and to 10.88% is also observed during corncob and sweet sorghum bagasse alkaline pretreatment [40]. It is expected that lignin removal will have a positive effect on biomass enzymatic degradation as the presence of lignin in the raw materials limits the enzymatic hydrolysis by impeding the cellulose accessibility [41]. As it can be seen, the removal of the non-cellulosic components of a polysaccharide nature also took place, resulting in the full removal of galactose, arabinose, and mannose and the partial removal of xylose. The removal of ash was observed.

The analysis of the solids after the alkaline pretreatment indicated that they contained a high percentage of cellulose (based on the glucose content, it was greater than 80%). It is expected that the cellulose in the solids after the alkaline pretreatment of the raw materials will most effectively transform into glucose.

According to the obtained results, the steam explosion pretreatment produced a minimal effect on the amount of glucose of both the apricot seed shells and walnut shells. The dissolution of part of low-molecular-weight polysaccharides took place. As it can be seen, the amount of dissolved hexoses, pentoses, and oligosaccharides was higher in liquid after the steam explosion pretreatment of the walnut shells due to their higher initial biomass in comparison with that of the apricot seed shells. The effect of the steam explosion pretreatment is not uniform with the amount of lignin in the pretreated materials. That of apricot seed shells increased probably due to reactions of condensation between the lignin, extractives of a different nature, and products of the sugar degradation [42], which agrees with experimental results of other studies [43,44]. At the same conditions, the amount of lignin in the walnut shell solid decreased; the same fact was observed during the steam explosion of spruce bark [45] and corn stalks [46]. Such differences in the behavior of lignin can be associated with the fact that steam explosion can cause the melting of lignin, and its partial depolymerization occurs through the cleavage of mainly ß-O-4 ether, resulting in the formation of alcohol derivatives, as well as condensation byproducts, the amount and composition of which largely depends on the type of feedstock [47].

### 2.3. Effect of Pretreatment on Hydrolysate Characterization

The effect of raw materials pretreatment with 1% H_2_SO_4_ at 180 °C in an agitated tank reactor and with the application of steam explosion on the hydrolysate composition is shown in Table 3. Hydrolysate after the alkaline pretreatment could not be analyzed because of the large amount of soluble and suspended substances that could not be recovered by centrifugation and membrane filtration.

As it can be seen, high glucose concentrations in the liquids after the acid pretreatment were obtained, which indicates a partial degradation of the cellulose during such a pretreatment. The amount of hemicellulosic sugars recovered in the liquids after the acid pretreatment (in form of monosaccharides) of both of the raw materials is much lower compared to those of other agricultural residues; for instance, the hemicellulosic sugars recovered after an olive tree pruning biomass pretreatment with diluted sulfuric acid was 78.5% [48]. The amount of recovered sugar in the liquid fractions obtained from the steam explosion pretreatment was very low, as can be seen in Table 3. A small amount of sugars recovered from the apricot seed shells and walnut shells is attributed to the complete depolymerization of hemicelluloses fraction into soluble monosaccharides and low-molecular-weight compounds during the acid pretreatment with 1% H_2_SO_4_ at 180 °C. Part of the acetyl groups in the hemicelluloses were cleaved, leading to the formation of acetic acid. Additionally, the formation of organic acids, such as formic and levulinic, as well as furfural and hydroxymethylfurfural, is detected as the result of monosaccharide degradation [49]. The amount of xylan, which is the largest constituent part of hemicelluloses, is the main indicator of the acid hydrolysis pretreatment. As it can be observed, xylan virtually disappeared in both of the raw materials when they were subjected to the acid pretreatment (Table 2).

Table 3 shows that liquid after the walnut shells acid pretreatment contained more inhibitors, which are the products of sugar degradation. The analysis shows that the liquid after the walnut shells pretreatment contained a high percentage of furfural and acetic acid (9.74 and 9.92% dry weight, respectively). Concerning the apricot seed shells, the liquid after the acid pretreatment contained far fewer inhibitor compounds due to the lower amount of hemicelluloses in the initial biomass.

The resulting liquid after the steam explosion pretreatment contained fewer inhibitors, indicating the preservation of sugars in the biomass.

### 2.4. Enzymatic Hydrolysis

**Figure 3:**
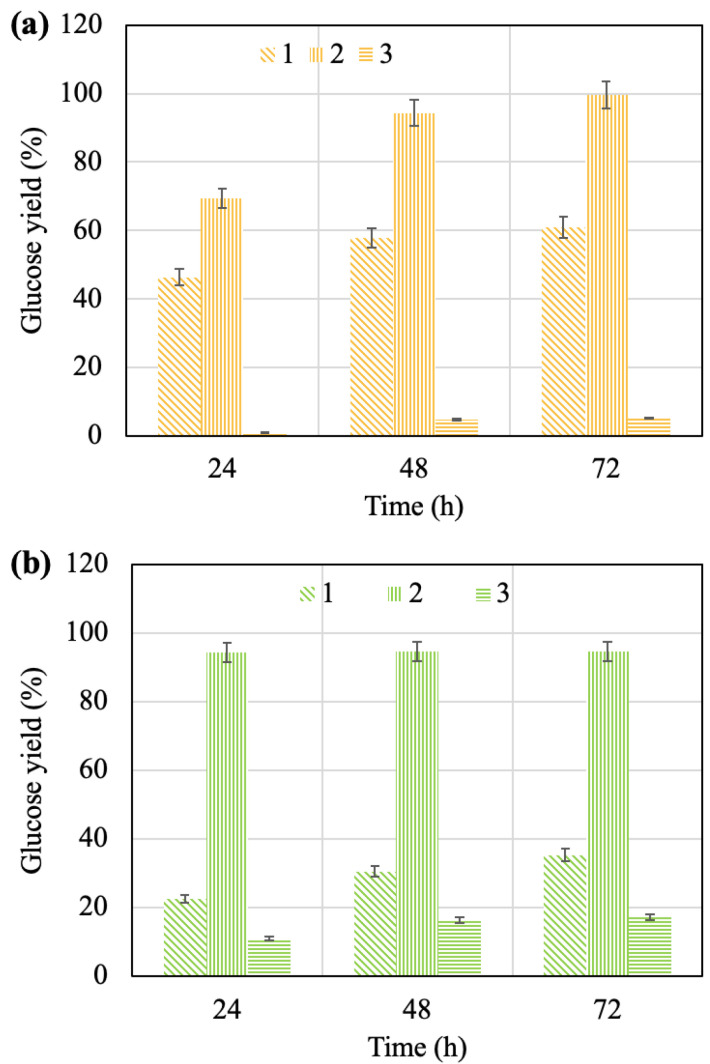
Effect of time on glucose yield during enzymatic hydrolysis of pretreated apricot seed shells (**a**) and walnut shells (**b**): 1—after acid pretreatment; 2—after alkaline pretreatment; 3—after steam explosion pretreatment.

The pretreated solids were submitted to enzymatic hydrolysis with the industrial cellulase complex called Cellic CTec2, and the effects of time on the efficiency of the process for the different solids were compared (Figure 3).

As expected, the cellulose hydrolysis yields increased with an increasing duration of the process for all of the samples, except for the solid after the alkaline pretreatment of the walnut shells, for which the maximum efficiency of hydrolysis was achieved in the first 24 h of contact, and a further increase in time almost did not lead to its change. As it can be seen, the alkaline and acid pretreatments are more efficient at increasing the yield of glucose than the steam explosion is. For the both of the samples of solids after the steam explosion pretreatment of the apricot seed shells and walnut shells, the maximum yields of glucose did not exceed 10 and 20%, respectively, while the yields for the solids after the acid pretreatment did not exceed 60 and 35%, respectively.

Maximum glucose yields (99.7% and 94.6%) from the solids were achieved with industrial cellulase enzymatic hydrolysis after the alkaline pretreatment of the raw materials. Hemicellulose removal and lignin removal from the raw materials during the acid and alkaline pretreatments probably created additional pores and sites in the biomass available for cellulolytic enzymes, which affected the cellulose. The effect is most pronounced with a low lignin content [50].

### 2.5. Solid Characterization after Enzymatic Hydrolysis

**Figure 4:**
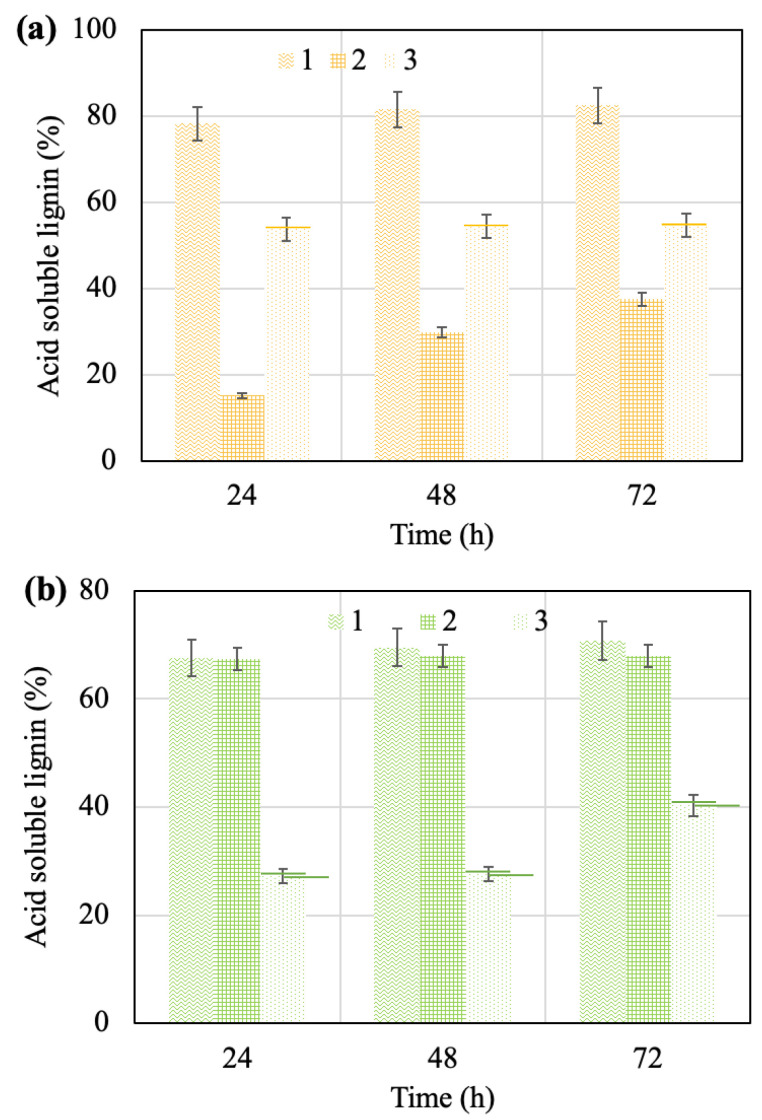
Effect of time of the process on amount of acid-soluble lignin in solids after hydrolysis of materials based on apricot seed shells (**a**) and walnut shells (**b**): 1—solid after acid pretreatment; 2—solid after alkaline pretreatment; 3—solid after pretreatment with steam explosion.

The maximum amount of lignin (82%) was achieved during the enzymatic hydrolysis of the solid after the acid pretreatment of the apricot seed shells due to the greater amount of lignin in the solid before the process (Figure 4a). In the case of walnut shells, the highest lignin values in the solids after enzymatic hydrolysis are around 70% for the cases of the acid and alkaline pretreated solids (Figure 4b). Samples subjected to 72 h of enzymatic hydrolysis were further analyzed.

According to Figure 4, the effect of time on the amount of acid-soluble lignin is limited, except for the apricot seed shells submitted to the alkaline pretreatment; in this case, an increase over time is observed. On the contrary, the amount remains virtually the same for the other pretreatments with this raw material and also for walnut shells in all of the cases.

**Figure 5:**
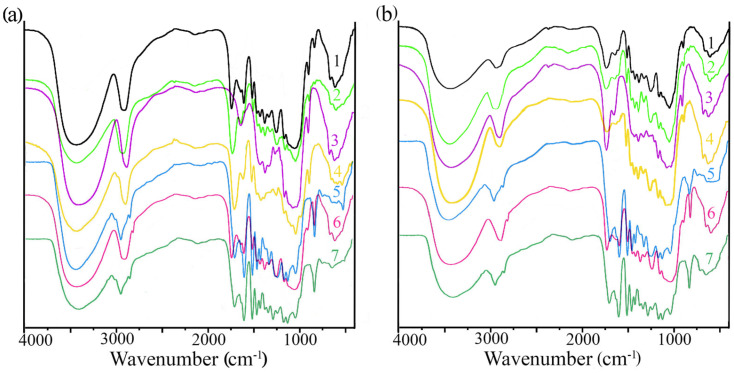
FTIR spectra for initial, pretreated and hydrolyzed apricot seed shells (**a**) and walnut shells (**b**): 1—initial materials; 2–4—solids, alkaline and steam explosion pretreatment, respectively; 5–7—respective solids after enzymatic hydrolysis with industrial cellulase Cellic CTec2 enzyme complex.

The FTIR spectra of all of the samples are characterized by the presence of different peaks for both the aromatic and polysaccharide components, but their intensities vary (Figure 5). The peaks between 3600–3000 cm^−1^ are presented in all of the spectra, and they are caused by the OH stretching vibration. The peaks between 2945–2855 cm^−1^ are attributed to C-H stretching vibration in all of the studied materials. The peaks at 1648, 1431, 1417, 1375, 1165, 1060, and 897 cm^−1^ are related to polysaccharides. The bands at 1605, 1509, and 1425 cm^−1^ correspond to the aromatic skeleton vibrations of lignin. Solids obtained thorough the alkaline pretreatment of the shells are characterized by an absence of bands at 1509 and 1425 cm^−1^ due to delignification. The bands at 1509 and 1425 cm^−1^ related to lignin become more intense in the sample obtained after the enzymatic hydrolysis of the solids after the steam explosion and acid pretreatment, and they also show that there is more lignin in these samples. The peak at 1249 cm^−1^ is related to C=C bonds in the lignin aromatic rings and C-O-C stretching in the hemicelluloses. This peak is less pronounced in the pretreated solids, which indicates the removal of hemicelluloses. The main difference between the FTIR spectra of the initial materials, the pretreated solids, and the solids after the hydrolysis is the increase in the intensity of the bands related to lignin (1509 and 1425 cm^−1^), which confirms that the resulting solids contain a large amount of lignin.

**Figure 6:**
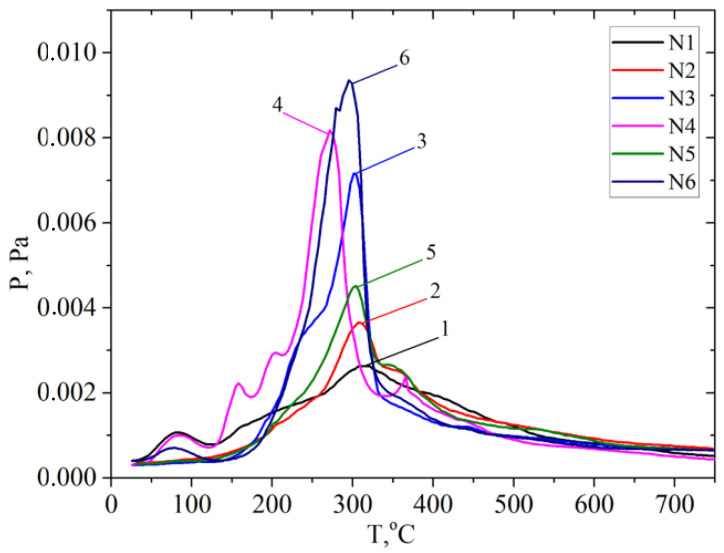
P/T curves of vapor pressure of gaseous products measured at temperature of pyrolysis: solids after hydrolysis of materials based on apricot seed shells (N1–3) and walnut shells (N4–6): 1 and 4—solid after alkaline pretreatment; 2 and 5—solid after acid pretreatment; 3 and 6—solid after pretreatment with steam explosion.

The samples were analyzed using temperature-programmed desorption mass spectrometry (TPD-MS). An analysis of the P/T curves of the pressure of the volatile pyrolysis products against the temperature of the sample shows significant differences from the behavior of the P/T curves for the studied samples depending on both the type of biomass and the type of pretreatment, as shown in Figure 6.

**Figure 7:**
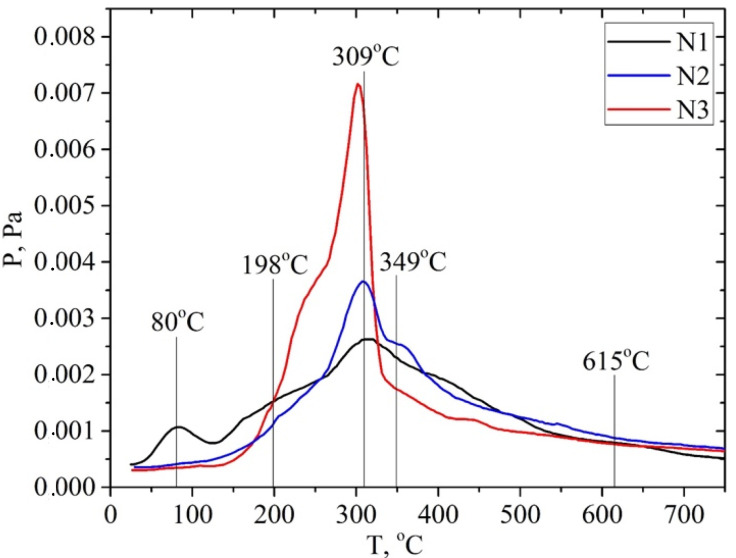
P/T curves of vapor pressure of gaseous products measured at temperature of pyrolysis: solids after hydrolysis of materials based on apricot seed shells: N1—solid after alkaline pretreatment; N2—solid after acid pretreatment; N3—solid after pretreatment with steam explosion.

**Figure 8:**
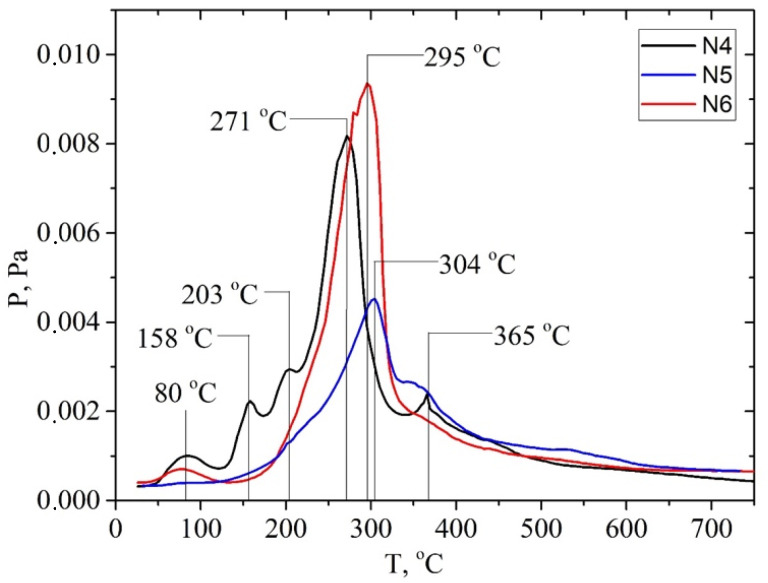
P/T curves of vapor pressure of gaseous products measured at temperature of pyrolysis: solids after hydrolysis of materials based on walnut shells: N4—solid after alkaline pretreatment; N5—solid after acid pretreatment; N6—solid after pretreatment with steam explosion.

A comparison of P/T curves for the apricot samples shows that the highest intensity of the desorption of pyrolysis products is observed for the sample after the pretreatment with a steam explosion (Figure 7). In this case, the intensity of the peak of desorption of pyrolysis products increases most significantly at a temperature of Tmax~300 °C. The intensity of this TPD peak for the sample apricot seed shells after the pretreatment with steam explosion is 2–3 times higher than the intensity for the samples after the acid and alkaline pretreatments. The same pattern is observed for the materials based on the walnut shells (Figure 8). Probably, such a treatment leads to the efficient depolymerization of lignocellulose.

**Figure 9:**
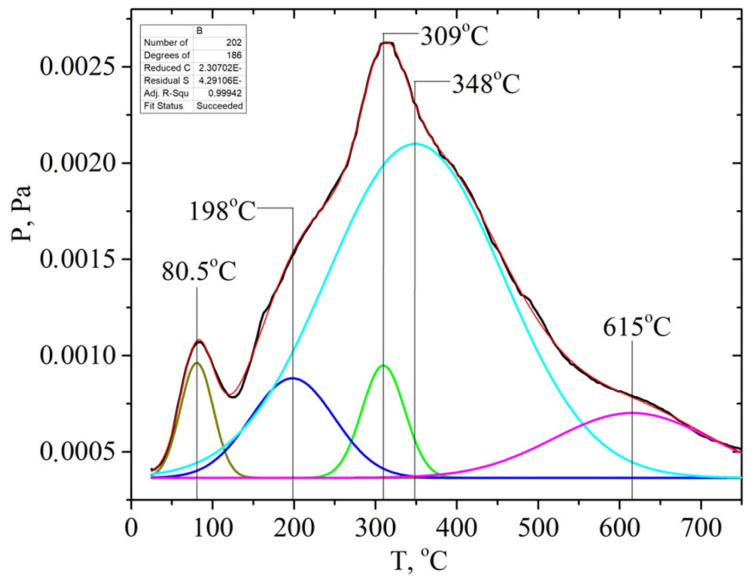
Deconvolution of the P/T curve for the solid based on apricot seed shells after alkaline pretreatment into separate Gaussians. Cellulose: apricot seed shell 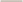; walnut shell 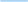; hemicellulose: apricot seed shell 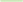; walnut shell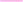. In addition, the ion with m/z 60 ([H_2_C=C(OH)_2_]^+^) is the most intense one in the mass spectrum of acetic acid. Hemicellulose pyrolysis is accompanied by the intensive desorption of acetic acid due to the elimination of acetyl groups. Therefore, according to the intensity of peaks on the TPD curve for the ion with m/z 60 at ~200 °C (150–250 °C), one can identify the relative amount of hemicelluloses in the biomass samples, and according to the intensity of the peak at ~300 °C (250–350 °C), one can identify the relative amount of cellulose, as shown in Figures S1–S6 (see SM file). The TPD MS data are in good agreement with the data in Table 3. The highest peak intensity on the TPD curve for m/z 60 at around ~200 °C is observed for the samples of apricot seed shells and walnut shells after the pretreatment with steam explosion (Figures S3 and S6). The lowest intensities are observed for the samples of apricot seed shells and walnut shells after the acid pretreatment (Figures S2 and S5), since acid hydrolysis leads to the almost complete dissolution and removal of hemicellulose from the biomass. In addition, the acid treatment leads to the hydrolysis of acetyl groups with the formation of acetic acid. This process can be used in “green technologies” to produce renewable bio-based acetic acid.

The P/T curve has overlapping peaks that correspond to the release of gaseous products due to thermal transformations of individual components of lignocellulose—hemicellulose, cellulose, and lignin. Decomposing the P/T curve into separate Gaussians makes it possible to identify the main stages of pyrolysis at 81, 198, 309, 348, and 615 °C, etc., (Figure 9).

It is known that the decomposition of the main components of plant biomass—hemicellulose, cellulose, and lignin—occurs at specific temperature ranges and is characterized by the temperatures of the maximum desorption rate, Tmax. Pyrolysis begins with the decomposition of the carbohydrate components of the biomass at a temperature of >150 °C, namely hemicellulose. The polymer chain of hemicellulose consists predominantly of monosaccharide five-membered xylose cycles, which are less stable than the six-membered glucose cycles are that make up cellulose. Therefore, the decomposition of cellulose is characterized by a higher Tmax~300 °C than that of hemicelluloses Tmax~200 °C. The most intense marker ion in the mass spectra of carbohydrate pyrolysis products is a fragment ion with m/z 60 (HOCHCHOH^+^) [51].

The thermal degradation of the lignin polymer chain occurs by the formation of many aromatic compounds. Previously, we found that lignin pyrolysis proceeds in two main stages [52]. Stage I is due to thermal transformations of external phenol–propanoic blocks. It is characterized by the temperature of the maximum desorption rate at about Tmax~320 ± 25 °C. It proceeds in the range of 220–440 °C depending on the type of biomass and its pretreatment (Figures S1–S6). The alkali pretreatment shifts the Tmax of this pyrolysis stage towards lower temperatures. The main pyrolysis products formed during stage I are syringol (m/z 154), methylguaiacol (m/z 138), 4-vinylpyrocatechol (m/z 136), guaiacol (m/z 124), pyrocatechol (m/z 110), phenol (m/z 94), etc.

Pyrolysis stage II is due to the decomposition of the internal phenol–propanoic blocks of lignin, which are interconnected by stronger chemical bonds. In this case, products with a lower molecular weight are formed: o-cresol or p-cresol (m/z 107, 108), phenol (m/z 94), toluene (m/z 91), benzene (m/z 78), etc. This process can be used in “green technologies” to produce renewable bio-based aromatic compounds.

### 2.6. Comparison with Previous Studies

A few works have been found in which fermentable sugars are produced from walnut shells; Table 4 shows some of these.

In the first of these works, a more complex two-stage process was used: first, a p-TsOH pretreatment, and then an H_2_O_2_ treatment and enzymatic hydrolysis with the same enzyme as that which was used in our work, Cellic CTec 2, but at a higher dose (40 FPU/g cellulose), achieving a hydrolysis yield of 94.4% [53], which is similar to what we have obtained when the alkaline pretreatment was performed. In the second work, a pretreatment with HNO_3_ and subsequent enzymatic hydrolysis with another enzyme ZSL cellulose (40 FPU/g solid) is carried out, reaching a hydrolysis yield of 80% [54], which is higher than that which was obtained when H_2_SO_4_ was used in our work, approx. 60%, but using a much lower dose of the enzyme than that which we used (15 FPU/g solid). In the last of the works, a pretreatment with a deep eutectic solvent was carried out, but the hydrolysis yield was low (16.94%) [55], as also happened in our case when we were using steam explosion. Table 4 shows more products that have been obtained from walnut shells, such as xylooligosaccharides, lignin, lignin nano-particles, and cellulose nanocrystals. In the case of the production of xylooligosaccharides, different strategies have been proposed. In one of the cases, the researchers proposed a first stage of delignification with NaClO_2_-CH_3_COOH and a second stage of alkaline extraction to recover the hemicelluloses that will later be enzymatically hydrolyzed [56], and on the other hand, the use of a single hydrothermal pretreatment stage [57]. In this latest work, lignin and cellulose nanocrystals are also produced after a second crganosolv delignification stage. To improve the recovery of lignin in another work, sequential organosolv delignification (3n) has been proposed, achieving close to 70% recovery of the initial lignin compared to less than 60% [58].

In the case of apricot seed shells, no previous reports have been found in the reviewed bibliography in which this raw material was used to produce sugars. This raw material has been used mainly to produce biomaterials such as nanocarbon, lignin-derived activated carbon, or cellulose basic-ion exchangers (Table 4). To produce these biomaterials, different processes have been used, for example, in the case of the production of the biomaterial which was used as a biosorbent, the apricot seed shells were subjected to a two-stage process, first, with NH_4_OH. and then, they were phosphorylated in an aqueous solution [59]. In the case of the production of cellulose basic-ion exchangers, a two-stage process has also been used, first, a treatment with ammonium, and then, amination using pyridine [60]. For the production of nanocarbons, simpler processes have been used in a single stage, including some more traditional ones using KOH [61] or H_3_PO_4_/KOH [62] for carbonization, and other ones that are more innovative such as “sol-gel” technology, with the use of catalysts of the type (CuO)x*(CoO)y*(NiO)z*(Fe_2_O_3_)k*(MoO_3_)m/HSZ [63].

**Table 4:** Process steps used to produce different products from walnut shell and apricot seed shells.

Process Steps			Products	Ref.
Step-1	Step-2	Enzymatic Hydrolysis		
Walnut shell				
p-TsOH pretreatment	H_2_O_2_ pretreatment	Cellic CTec 2 (40 FPU/g cellulose)	Fermentable sugars	[53]
HNO_3_ pretreatment		ZSL cellulose (40 FPU/g solid)	Fermentable sugars	[54]
Deep eutectic solvent		Cellulase from *Trichoderma viride* (Novozymes)	Lignin nano-particles Fermentable sugars	[55]
NaClO_2_-CH_3_COOH delignification	Alkaline extraction	Commercially available endo-1,4-β-xylanase	Xylooligosaccharides	[56]
Hydrothermal pretreatment	Organosolv delignification		Xylooligosaccharides Lignin Cellulose nanocrystals	[57]
Sequential organosolv delignification (3n)	Hydrothermal treatment		Lignin Cellulose nanocrystals	[58]
Apricot seed shells				
NH_4_OH pretreatment	Phosphorylated in an aqueous solution		Biosorbent	[59]
“Sol-gel” technology. (CuO)x*(CoO)y*(NiO)z*(Fe_2_O_3_)k*(MoO_3_)m/HSZ based catalyst			Nanocarbon	[60]
H3PO4/KOH carbonization			Lignin-derived activated carbon	[61]
NH_4_OH pretreatment	Aminated using pyridine		Cellulose basic-ion exchangers	[62]
KOH carbonization			Nanocarbon	[63]

### 2.7. Mass Balance

**Figure 10:**
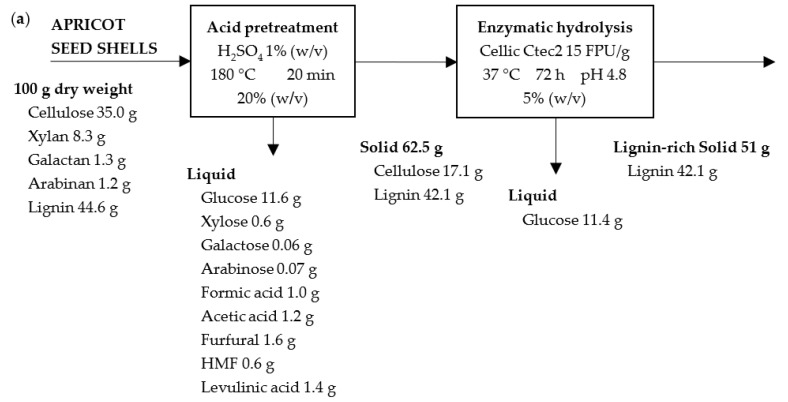

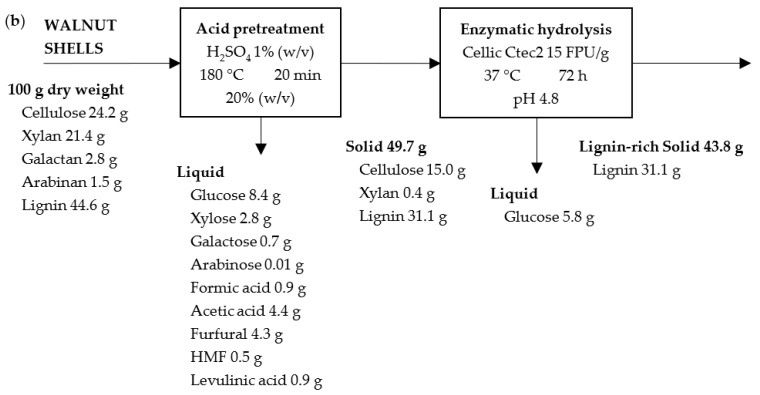
Mass balance for acid pretreatment and enzymatic hydrolysis of apricot seed shells (**a**) and walnut shells (**b**).

**Figure 11:**
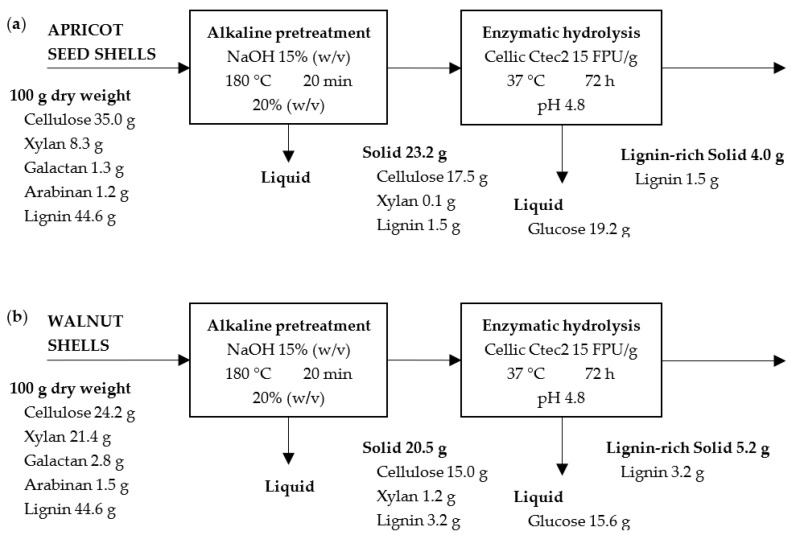
Mass balance for alkaline pretreatment and enzymatic hydrolysis of apricot seed shells (**a**) and walnut shells (**b**).

**Figure 12:**
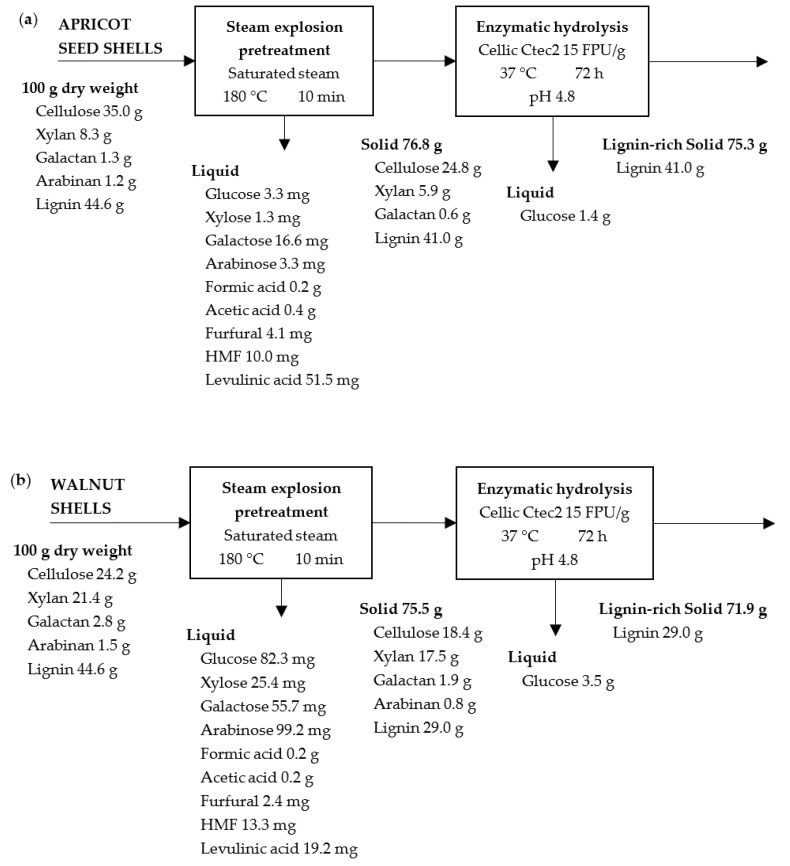
Mass balance for steam explosion pretreatment and enzymatic hydrolysis of apricot seed shells (**a**) and walnut shells (**b**).

Mass balance was built for each material and pretreatment type, and it is shown in Figure 10, Figure 11 and Figure 12. In all of the cases, the results are given for 100 g of raw material for ease of comparison. In the pretreatment stage, the type of pretreatment is the difference between the scheme (i.e., acid, alkaline, and steam explosion ones). As can be seen, in each scheme 62.5 g, 23.2 g, and 76.8 g of pretreated solids from the apricot seed shells and 49.7 g, 20.5 g, and 75.5 g of pretreated solids from then walnut shells were generated. The higher solid yield after steam explosion can be attributed to the fact that steam explosion is a hydrothermal pretreatment used to disrupt the bonding between components in a lignocellulosic complex through the use of high-pressure steam for some time and rapid explosive decompression. This is a type of physicochemical treatment, as high-pressure steam break the lignocellulose structure resulting in the partial degradation of hemicellulose and the removal of extractives. During the acid pretreatment, mostly the hydrolysis of polysaccharides (10.7% in initial apricot seed shells and 25.7% in walnut shells), as well as the removal of extractives, took place. During the alkali pretreatment, mostly the oxidation and removal of lignin (nearly 44.6% in initial material) took place. Both of the processes resulted in a lower yield of solids in comparison to that of the steam explosion experiment.

The second scheme (Figure 11) is characterized by the production of liquids with the most dissolved substances. In the first scheme (Figure 10), complete hemicellulose hydrolysis was achieved for the apricot seed shells, but for walnut shells, the resulting solid contains a small amount of xylose. At the same time, both of the solids are enriched in lignin. In the second scheme, the amount of lignin was reduced significantly for both the raw materials. The same happened regarding the amount of hemicellulose. In the third scheme (Figure 12), the maximum yield of the pretreated solid was obtained. The third scheme is characterized by maintaining the maximum amount of lignin and hemicellulosic sugars in the pretreated materials, resulting in the high solid yield (Figure 12).

The pretreated solids were submitted to enzymatic hydrolysis with an industrial cellulase Cellic CTec2 enzyme complex in the presence of β-glucanase. In the second stage, 11.4 g and 5.8 g of glucose after the acid pretreatment, 19.2 g and 15.6 g of glucose after the alkaline pretreatment, and 1.4 g and 3.5 g of glucose after the steam explosion pretreatment with the apricot seed shells and walnut shells, respectively, were generated. A complete cellulose conversion to glucose was not achieved, but the alkaline pretreatment with 15% NaOH was the most optimal type for the pretreatment of both of the raw materials, resulting in the highest glucose yields. The possible reason for this could be the removal of lignin and hemicellulosic sugars.

## 3. Materials and Methods

### 3.1. Materials

Shells of walnuts (*Juglans regia* L.) and shells of apricot seed (*Prunus armeniaca*) from the Odessa region of Ukraine were used as raw materials. The shells were milled. For this purpose, a laboratory hammer mill (Retsch, SM 100, Haan, Germany) was used. A fraction of material passed through a 1 mm screen was kept in a sealed plastic bag and used in the experiments.

Industrial cellulase Cellic CTec2 enzyme complex and β-glucanase were supplied by Novozymes A/S, Bagsværd, Denmark. The chemicals were purchased from Sigma-Aldrich (Darmstadt, Germany).

### 3.2. Biomass Pretreatment

The fraction of milled shells that passed through the 1 mm screen was used for the pretreatment experiments (dilute acid and alkali solution, as well as steam explosion), and the resulting pretreated solids were submitted to enzymatic hydrolysis. The pretreatments with 1% H_2_SO_4_ and 15% NaOH (*w*/*v*) were performed in a 1 L agitated tank reactor (Parr Instrument Company, Moline, IL, USA). The biomass loading inside the laboratory reactor was 20% (*w*/*v*). The targeted temperature was 180 °C, and the heating time was 40 min. As the targeted temperature was reached, the processing time was 20 min. Agitation at 300 rpm was applied. At the end of the acid and alkali pretreatment, we stopped heating it, and the reactor was cooled with tap water to room temperature, and the solids were filtered and washed with deionized water. The pretreated solids were oven dried at 50 °C for 48 h to reach a moisture content of 3%.

The steam explosion treatment of the shells without initial impregnation was carried out in a custom-built batch pilot unit in a 4 L reaction vessel. To perform the experiment, the vessel reactor was loaded with 500 g of sample, and then the reactor was heated with saturated steam to 180 °C. The process time was 10 min. Then, the reaction vessel was rapidly depressurized to an atmospheric pressure.

### 3.3. Enzymatic Hydrolysis

The pretreated solids were used as substrates for a further enzymatic hydrolysis. The prepared materials were hydrolyzed with an industrial cellulase Cellic CTec2 enzyme complex. The cellulase enzyme loading was exactly 15 FPU per 1 g of substrate. To provide the necessary β-glucosidase activity of cellulases, β-glucanase was added. The pH of the solution was adjusted to 4.8 with 0.05 M sodium citrate buffer, and then, the enzymes were added to the lignocellulosic substrate (5% *w*/*v* dry basis). During the experiment, the total working volume was 25 mL in 100 mL Erlenmeyer flasks. All the experiments were performed in triplicate reaction flasks. The flasks were kept at 50 °C for 72 h in an orbital shaker at 150 rpm (Certomat-R, B-Braun, Göttingen, Germany). To evaluate the efficiency of hydrolysis, samples of 1 mL were taken at 24, 48, and 72 h. The samples were centrifuged for 10 min at 10,000× *g* rpm using Sigma 1–14 Centrifuge (Osterode am Harz, Germany), and then used for the determination of glucose and xylose concentrations by high-performance liquid chromatography (HPLC, Agilent Technologies, Palo Alto, CA, USA).

### 3.4. Raw Material, Residual Solid, and Liquid Fraction (Hydrolysate) Characterization

The chemical composition of the biomass was investigated according to the methodology from NREL (National Renewable Energy Laboratory, Golden, CO, USA) [64,65,66,67]. The pretreated solids yields were determined gravimetrically. The liquid fraction was also analyzed and previously filtered through 0.45 μm nylon membranes. The monomer sugars, such as glucose, xylose, mannose, galactose, and arabinose, and inhibitor composition, such as acetic acid, formic acid, furfural, and hydroxymethylfurfural (HMF), of the liquid fraction after acid and steam explosion pretreatments were determined by HPLC using an Agilent Technologies 1200 series HPLC system (Santa Clara, CA, USA), which was equipped with ICSep ICE-COREGEL 87H3 column. The column was operating at 65 °C, and 5 mM sulfuric acid was used as the mobile phase (0.6 mL/min).

The amount of polysaccharide, acid-insoluble lignin, and acid-soluble lignin in the solids was also determined according to methodology from NREL. The hydrolysates after the pretreatment were also analyzed, and the amount of sugars were determined. Glucose and xylose recoveries were calculated as the ratio of sugars that remains in the pretreated solids and liquid fractions to sugars present in the raw material. Monomeric and oligomeric sugars in the liquid fractions after pretreatments were investigated by HPLC, as well as the amount of formic acid, acetic acid, furfural, hydroxymethylfurfural (HMF), and levulinic acid.

Glucose and xylose concentrations in the sample supernatant were determined by HPLC using the liquid chromatograph with a refractive index detector (CARBOSep CHO-782 Pb, Transgenomic, Inc., Omaha, NE, USA) with ultra-pure water as an eluent. The flow rate was 0.6 mL/min, and the column temperature was 70 °C.

Fourier transform infrared (FTIR) spectroscopy using a Perkin-Elmer Spectrum 100 FTIR spectrometer (Golden gate from Graseby Specac LTD, Kent, England) was used to evaluate structural changes in the solids. All of the materials were analyzed in the 4000–500 cm^−1^ range at a resolution of 4 cm^−1^.

All of the experiments and determinations were carried out in triplicate. Relative standard deviations are below 5%.

### 3.5. Temperature-Programmed Desorption Mass Experiments

Temperature-programmed desorption spectrometry (TPD MS) was performed using a monopole mass spectrometer (MKh-7304A, Sumy, Ukraine) with electron impact ionization adapted to study pyrolysis kinetics [68,69,70]. About 8 mg of biomass samples were used for each experiment. TPD measurements were performed at a constant rate of 0.168 °C/s. Mass spectra, TPD curves, and the P/T pressure–temperature curves (P—the pressure of volatile pyrolysis products; T—temperature of the sample) were created and analyzed in a computer-based data acquisition and processing step.

## 4. Conclusions

The agricultural residues largely available in Ukraine such as apricot seed shells and walnut shells, which are underutilized, have a low cost, and lack applications, can be regarded as suitable renewable raw materials for multiproduct biorefinery. An evaluation of the influence of the pretreatments on raw materials shows that alkaline pretreatment is characterized by minimal solid recovery due to the removal of large amounts of hemicelluloses and lignin. Acid pretreatment is effective at solubilizing hemicellulose, while steam explosion has a less destructive effect on lignin and hemicellulose. The removal of the mentioned components exposes cellulose, resulting in an increase in its accessibility. Alkaline pretreatment allows us to achieve a high glucose yield. Both of the materials are a great alternative and prospective source of fermentable sugars, which indicates their potential in biofuel production (energy independence) for other bioproducts such as bioplastics. The results show that the alkali pretreatment is more advantageous, not only because, overall, a higher glucose yield is obtained, but also because it is industrially more feasible as lignin can be easily precipitated from the spent liquor and processed again into value-added products, as well as sodium hydroxide, which can be easily recovered and returned to the technological process. The proposed application represents an innovative method of disposal on the one hand, and the possibility of producing renewable compounds on the other side, thus contributing to the development of a circular bioeconomy.

Future work will focus on the conversion of sugars into valuable compounds as a result of a fermentation process; for these studies, attention will be paid especially to the formation of potential inhibitors of microorganism actions due to the conditions of the pretreatment, and a compromise between a high sugar yield and minimum degradation will be procured. In addition to sugars, a relevant future development will be the characterization of lignins in terms of average molecular weight, average molecular number, and polydispersity index, among others, with the general objective of taking full advantage of these renewable feedstocks.

## Figures and Tables

**Table 1:** Chemical composition of apricot seed shells and walnut shells (% dry weight).

Components	Apricot Seed Shells	Walnut Shells
Extractives	9.97 ± 0.51	11.41 ± 2.23
Cellulose	35.01 ± 0.42	24.19 ± 0.68
Hemicellulose Xylan Galactan Arabinan Mannan	10.77 ± 0.14 8.33 ± 0.09 1.28 ± 0.05 1.16 ± 0.02 -	25.68 ± 1.70 21.35 ± 1.83 2.78 ± 0.07 1.55 ± 0.01 -
Lignin Acid-Soluble Lignin Acid-Insoluble Lignin	44.55 ± 1.05 1.12 ± 0.01 43.43 ± 1.04	44.63 ± 1.01 1.14 ± 0.01 43.49 ± 1.00
Ash Acid-insoluble ash	0.23 ± 0.01 -	0.37 ± 0.01 0.12 ± 0.00
Acetyl groups Acetic acid	0.85 ± 0.02 1.21 ± 0.03	3.87 ± 0.09 5.53 ± 0.12

**Table 2:** Solid characterization after acid, alkaline, and steam explosion pretreatment of shells.

Material	Sugars Content (%)					Acid-Insoluble Ash (%)
	Glucan	Xylan	Galactan	Arabinan	Mannan	
Acid pretreatment						
Apricot seed shells	27.43 ± 0.29	-	-	-	-	-
Walnut shells	30.30 ± 0.64	0.90 ± 0.06	-	-	-	0.05 ± 0.00
Alkaline pretreatment						
Apricot seed shells	75.58 ± 1.14	3.59 ± 0.28	-	-	-	-
Walnut shells	73.38 ± 1.58	5.57 ± 0.09	-	-	-	-
Steam explosion pretreatment						
Apricot seed shells	32.32 ± 0.64	7.75 ± 0.29	0.57 ± 0.09	-	-	-
Walnut shells	24.35 ± 0.12	23.25 ± 0.39	2.58 ± 0.06	1.24 ± 0.05	-	-

**Table 3:** Hydrolysate characterization after acid and steam explosion pretreatment of raw materials.

Composition	Acid Pretreatment		Steam Explosion Pretreatment	
	Apricot Seed Shells	Walnut Shells	Apricot Seed Shells	Walnut Shells
Hemicellulosic sugars recovery (%)	4.21	10.91	2.02	5.24
Glucose recovery (%)	37.16	35.67	0.02	3.52
Sugars content (g/L)				
Glucose	27.86 ± 0.08	18.98 ± 0.07	0.02 ± 0.00	0.68 ± 0.01
Xylose	1.35 ± 0.02	6.33 ± 0.02	0.08 ± 0.00	0.21 ± 0.01
Galactose	0.17 ± 0.00	1.55 ± 0.01	0.10 ± 0.00	0.46 ± 0.01
Arabinose	-	0.19 ± 0.00	0.02 ± 0.00	0.82 ± 0.01
Mannose	0.17 ± 0.00	-	-	-
Inhibitors (g/L)				
Formic acid	2.45 ± 0.01	1.96 ± 0.02	1.42 ± 0.01	1.87 ± 0.02
Acetic acid	2.91 ± 0.01	9.92 ± 0.03	2.16 ± 0.02	1.59 ± 0.01
Furfural	3.74 ± 0.02	9.74 ± 0.03	0.00 ± 0.00	0.02 ± 0.00
Hydroxymethylfurfural	1.47 ± 0.01	1.06 ± 0.01	0.06 ± 0.00	0.11 ± 0.00
Levulinic acid	3.31 ± 0.02	2.05 ± 0.02	0.31 ± 0.01	0.16 ± 0.01

## Data Availability

Not applicable.

## Supplementary Materials

The following supporting information can be downloaded at: https://www.mdpi.com/article/10.3390/molecules28031455/s1, Figure S1: TPD curves for ions with m/z 154, 138, 136, 124, 110, 109, 108, 107, 94, 91, 78, 60, and 55 obtained via pyrolysis of the solid based on apricot seed shells after alkaline pretreatment; Figure S2: TPD curves for ions with m/z 154, 138, 136, 124, 110, 109, 108, 107, 94, 91, 78, 60, and 55 obtained via pyrolysis of the solid based on apricot seed shells after acid pretreatment; Figure S3: TPD curves for ions with m/z 154, 138, 136, 124, 110, 109, 108, 107, 94, 91, 78, 60, and 55 obtained via pyrolysis of the solid based on apricot seed shells after pretreatment with steam explosion; Figure S4: TPD curves for ions with m/z 154, 138, 136, 124, 110, 109, 108, 107, 94, 91, 78, 60, and 55 obtained via pyrolysis of the solid based on walnut shells after alkaline pretreatment; Figure S5: TPD curves for ions with m/z 154, 138, 136, 124, 110, 109, 108, 107, 94, 91, 78, 60, and 55 obtained via pyrolysis of the solid based on walnut shells after acid pretreatment; Figure S6: TPD curves for ions with m/z 154, 138, 136, 124, 110, 109, 108, 107, 94, 91, 78, 60, and 55 obtained via pyrolysis of the solid based on walnut shells after pretreatment with steam explosion.

## References

[1] Miller R.G., Sorrell S.R. (2014). The future of oil supply. Philos. Trans. A Math. Phys. Eng. Sci..

[2] Bay M.S., Karimi K., Nasr Esfahany M., Kumar R. (2020). Structural modification of pine and poplar wood by alkali pretreatment to improve ethanol production. Ind. Crop. Prod..

[3] Grassi M.C.B., Pereira G.A.G. (2019). Energy-cane and RenovaBio: Brazilian vectors to boost the development of Biofuels. Ind. Crop. Prod..

[4] Patni N., Pillai S.G., Dwivedi A.H. (2013). Wheat as a promising substitute of corn for bioethanol production. Procedia Eng..

[5] Akao S., Yasutake D., Kondo K., Nagare H., Maeda M., Fujiwara T. (2018). Effects of cultivation period on catch crop chemical composition and potential for bioenergy production. Ind. Crop. Prod..

[6] Chavan S., Gaikwad A. (2020). Optimization of enzymatic hydrolysis of bamboo biomass for enhanced saccharification of cellulose through Taguchi orthogonal design. J. Environ. Chem. Eng..

[7] Duruyurek M., Düşgün C., Gulhan M., Selamoglu Z. (2015). Production of Bioethanol from Waste Potato. Turkish J. Agric.-Food Sci. Tech..

[8] Nashiruddin N.I., Mansor A.F., Rahman R.A., Ilias R.M., Yussof H.W. (2020). Process parameter optimization of pretreated pineapple leaves fiber for enhancement of sugar recovery. Ind. Crop. Prod..

[9] Kartel M., Galysh V. (2017). New composite sorbents for Caesium and Strontium ions sorption. Chem. J. Mold..

[10] Deykun I., Halysh V., Barbash V. (2018). Rapeseed straw as an alternative for pulping and papermaking. Cellulose Chem. Technol..

[11] Halysh V., Trembus I., Deykun I., Ostapenko A., Nikolaichuk A., Ilnitska G. (2018). Development of effective technique for the disposal of the Prunus Armeniaca seed shells. East-Eur. J. Enterp. Technol..

[12] Mahari W.A.V., Waiho K., Fazhan H., Necibi M.C., Hafsa J., Mrid R.B., Fal S., Arrousi H.E., Peng W., Tabatabaei M. (2021). Progress in valorisation of agriculture, aquaculture and shellfish biomass into biochemicals and biomaterials towards sustainable bioeconomy. Chemosphere.

[13] Peinemann J.C., Pleissner D. (2020). Continuous pretreatment, hydrolysis, and fermentation of organic residues for the production of biochemicals. Bioresour. Technol..

[14] Gilna P., Lynd L.R., Mohnen D., Davis M.F., Davison B.H. (2017). Progress in understanding and overcoming biomass recalcitrance: A BioEnergy Science Center (BESC) perspective. Biotechnol. Biofuels.

[15] Zhang Y., Huang M., Su J., Hu H., Yang M., Huang Z., Chen D., Wu J., Feng Z. (2019). Overcoming biomass recalcitrance by synergistic pretreatment of mechanical activation and metal salt for enhancing enzymatic conversion of lignocellulose. Biotechnol. Biofuels.

[16] Meng X., Pu Y., Yoo C.G., Li M., Bali G., Park D.Y., Gjersing E., Davis M.F., Muchero W., Tuskan G.A. (2017). An in-depth understanding of biomass recalcitrance using natural poplar variants as the feedstock. Chem. Sus. Chem..

[17] Kucharska K., Rybarczyk P., Hołowacz I., Łukajtis R., Glinka M., Kamiński M. (2018). Pretreatment of lignocellulosic materials as substrates for fermentation processes. Molecules.

[18] Momayez F., Karimi K., Karimi S., Horváth I.S. (2017). Efficient hydrolysis and ethanol production from rice straw by pretreatment with organic acids and effluent of biogas plant. RSC Adv..

[19] Xing Y., Yu H., Zhu L., Jiang J. (2013). Efficient enzymatic hydrolysis of bamboo by pretreatment with steam explosion and alkaline peroxide. BioResources.

[20] Cutrim F.M., Ramos E.C.S.S., Abreu M.C.C., Godinho A.S., Maciel A.P., Mendonça C.J.S., Cavalcante K.S.B. (2019). A study of chemical composition and enzymatic hydrolysis of solid organic waste from Agrosilvopastoral systems. J. Braz. Chem. Soc..

[21] Kontogianni N., Barampouti E.M., Mai S., Malamis D., Loizidou M. (2019). Effect of alkaline pretreatments on the enzymatic hydrolysis of wheat straw. Environ. Sci. Pollut. Res..

[22] De Carvalho D.M., Sevastyanova O., Penna L.S., de Silva B.P., Lindström M.E., Colodette J.L. (2015). Assessment of chemical transformations in eucalyptus, sugarcane bagasse and straw during hydrothermal, dilute acid, and alkaline pretreatments. Ind. Crop. Prod..

[23] Romero-García J.M., Lama-Muñoz A., Rodríguez-Gutiérrez G., Moya M., Ruiz E., Fernández-Bolaños J., Castro E. (2016). Obtaining sugars and natural antioxidants from olive leaves by steam-explosion. Food Chem..

[24] Li G., He W., Yuan L. (2017). Aqueous ammonia pretreatment of sugar beet pulp for enhanced enzymatic hydrolysis. Bioprocess Biosyst. Eng..

[25] FAO—Food and Agriculture Organization Food and Agriculture Data. https://www.fao.org/faostat/en/#home.

[26] Halysh V., Trus I., Nikolaichuk A., Skiba M., Radovenchyk I., Deykun I., Vorobyova V., Vasylenko I., Sirenko L. (2020). Spent biosorbents as additives in cement production. J. Ecol. Eng..

[27] Halysh V., Sevastyanova O., de Carvalho D.M., Riazanova A.V., Lindström M.E., Gomelya M. (2019). Effect of oxidative treatment on composition and properties of sorbents prepared from sugarcane residue. Ind. Crop. Prod..

[28] Trembus I.V., Trophimchuk J.S., Galysh V.V. (2018). Preparation of pulp from sunflower stalks using peroxy acids. Voprosy Khimii i Khimicheskoi Tekhnologii.

[29] Trembus I., Hondovska A., Halysh V., Deykun I., Cheropkina R. (2022). Feasible Technology for Agricultural Residues Utilization for the Obtaining of Value-Added Products. Ecol. Eng. Environ. Technol..

[30] Yu Z., Jameel H., Chang H.M., Park S. (2011). The effect of delignification of forest biomass on enzymatic hydrolysis. Bioresour. Technol..

[31] Park J., Shin H., Yoo S., Zoppe J.O., Park S. (2015). Delignification of lignocellulosic biomass and its effect on subsequent enzymatic hydrolysis. BioResources.

[32] Corbett D.B., Kohan N., Machado G., Jing C., Nagardeolekar A., Bujanovic B.M. (2015). Chemical composition of apricot pit shells and effect of hot-water extraction. Energies.

[33] Queirós C., Cardoso S., Lourenço A., Ferreira J., Miranda I., Lourenço M., Pereira H. (2020). Characterization of walnut, almond, and pine nut shells regarding chemical composition and extract composition. Biomass Conv. Bioref..

[34] Pirayesh H., Khazaeian A., Tabarsa T. (2012). The potential for using walnut (*Juglans regia* L.) shell as a raw material for wood-based particleboard manufacturing. Compos. Part B—Eng..

[35] Wu Z., Hao H., Tu Y., Hu Z., Wei F., Liu Y., Zhou Y., Wang Y., Xie G., Gao C. (2014). Diverse cell wall composition and varied biomass digestibility in wheat straw for bioenergy feedstock. Biomass Bioenergy.

[36] Oka D., Kobayashi K., Isobe N., Ogawa Y., Yokoyama T., Kimura S., Kim U., Tokuyasu K., Wada M. (2013). Enzymatic hydrolysis of wood with alkaline treatment. J. Wood Sci..

[37] Siddhu M.A.H., Li W., He Y., Liu G., Chen C. (2019). Steam explosion pretreatment of rice straw to improve structural carbohydrates anaerobic digestibility for biomethanation. Environ. Sci. Pollut. Res..

[38] Benjamin Y., Cheng H., Görgens J.F. (2014). Optimization of dilute sulfuric acid pretreatment to maximize combined sugar yield from sugarcane bagasse for ethanol production. Appl. Biochem. Biotechnol..

[39] Kim J.S., Lee Y.Y., Kim T.H. (2016). A review on alkaline pretreatment technology for bioconversion of lignocellulosic biomass. Bioresour. Technol..

[40] Mafa M.S., Malgas S., Bhattacharya A., Rashamuse K., Pletschke B.I. (2020). The effects of alkaline pretreatment on agricultural biomasses (corn cob and sweet sorghum bagasse) and their hydrolysis by a termite-derived enzyme cocktail. Agronomy.

[41] Herbaut M., Zoghlami A., Habrant A., Falourd X., Foucat L., Chabbert B., Paës G. (2018). Multimodal analysis of pretreated biomass species highlights generic markers of lignocellulose recalcitrance. Biotechnol. Biofuels.

[42] Steinbach D., Kruse A., Sauer J., Storz J. (2020). Is Steam Explosion a Promising Pretreatment for Acid Hydrolysis of Lignocellulosic Biomass. Processes.

[43] Auxenfans T., Crônier D., Chabbert B., Paës G. (2017). Understanding the structural and chemical changes of plant biomass following steam explosion pretreatment. Biotechnol. Biofuels.

[44] He Q., Ziegler-Devin I., Chrusciel L., Ngwa Obame S., Hong L., Lu X., Brosse N. (2020). Lignin-first integrated steam explosion process for green wood adhesive application. ACS Sustain. Chem. Eng..

[45] Kemppainen K., Inkinen J., Uusitalo J., Nakari-Setälä T., Siika-aho M. (2012). Hot water extraction and steam explosion as pretreatments for ethanol production from spruce bark. Biores Technol..

[46] Wang X.T., Liu L.S. (2010). Steam Explosion Pretreatment Technique and Application in Biomass Conversion. Adv. Mater. Res..

[47] Singh J., Suhag M., Dhaka A. (2015). Augmented digestion of lignocellulose by steam explosion, acid and alkaline pretreatment methods: A review. Carbohydr. Polym..

[48] Díaz-Villanueva M., Cara-Corpas C., Ruiz-Ramos E., Romero-Pulido I., Castro-Galiano E. (2012). Olive tree pruning as an agricultural residue for ethanol production. Fermentation of hydrolysates from dilute acid pretreatment. Span. J. Agric. Res..

[49] Baadhe R.R., Potumarthi R., Mekala N.K. (2014). Influence of dilute acid and alkali pretreatment on reducing sugar production from corncobs by crude enzymatic method: A comparative study. Bioresour. Technol..

[50] Shimizu F.L., Monteiro P.Q., Ghiraldi P.H.C., Melati R.B., Pagnocca F.C., Souza W., Sant’Anna C., Brienzo M. (2018). Acid, alkali and peroxide pretreatments increase the cellulose accessibility and glucose yield of banana pseudostem. Ind. Crop. Prod..

[51] Lebedev A. (2003). Mass Spectrometry in Organic Chemistry.

[52] Palianytsia B., Kulik T., Dudik O., Cherniavska T., Tonkha O. Study of the thermal decomposition of some components of biomass by desorption mass spectrometry. Proceedings of the International Congress on Energy Efficiency and Energy Related Materials (ENEFM2013).

[53] Zhu J., Jiao N., Li H., Xu G., Zhang H., Xu Y. (2022). P-Toluenesulfonic Acid Combined with Hydrogen Peroxide-Assisted Pretreatment Improves the Production of Fermentable Sugars from Walnut (*Juglans regia* L.) Shells. Bioresour. Technol..

[54] Tan M., Ma L., Rehman M.S.U., Ahmed M.A., Sajid M., Xu X., Sun Y., Cui P., Xu J. (2019). Screening of Acidic and Alkaline Pretreatments for Walnut Shell and Corn Stover Biorefining using Two Way Heterogeneity Evaluation. Renew. Energy.

[55] Li H., Liang J., Chen L., Ren M., Zhou C. (2023). Utilization of Walnut Shell by Deep Eutectic Solvents: Enzymatic Digestion of Cellulose and Preparation of Lignin Nanoparticles. Ind. Crop. Prod..

[56] Cebin A.V., Ralet M., Vigouroux J., Karača S., Martinić A., Komes D., Bonnin E. (2021). Valorisation of Walnut Shell and Pea Pod as Novel Sources for the Production of Xylooligosaccharides. Carbohydr. Polym..

[57] Morales A., Labidi J., Gullón P. (2021). Hydrothermal Treatments of Walnut Shells: A Potential Pretreatment for Subsequent Product Obtaining. Sci. Total Environ..

[58] Morales A., Labidi J., Gullón P. (2022). Integral Valorisation of Walnut Shells Based on a Three-Step Sequential Delignification. J. Environ. Manag..

[59] Yelatontsev D. (2023). Production of Versatile Biosorbent Via Eco-Friendly Utilization of Non-Wood Biomass. Chem. Eng. J..

[60] Yelatontsev D.A., Mukhachev A.P. (2020). Synthesis and Properties of Ion Exchangers Derived from Non-Wood Cellulose. ChemChemTech.

[61] Saha D., Taylor B., Alexander N., Joyce D.F., Faux G.I., Lin Y., Shteyn V., Orkoulas G. (2018). One-Step Conversion of Agro-Wastes to Nanoporous Carbons: Role in Separation of Greenhouse Gases. Bioresour. Technol..

[62] Wang L., Feng X., Li X., Ma H., Wu J., Chen Y., Zhou J. (2022). Valorization of Lignin: Application of Lignin-Derived Activated Carbon in Capacitors and Investigation of its Textural Properties and Electrochemical Performance. Diam. Relat. Mat..

[63] Xolmirzayeva H.N., Fayzullayev N.I. (2022). Obtaining Nanocarbon from Local Raw Materials and Studying its Textural and Sorption Properties. Int. J. Eng. Trends Technol..

[64] NREL—National Renewable Energy Laboratory Determination of Structural Carbohydrates and Lignin in Biomass. https://www.nrel.gov/docs/gen/fy13/42618.pdf.

[65] NREL—National Renewable Energy Laboratory Ash in Biomass. https://www.nrel.gov/docs/gen/fy08/42622.pdf.

[66] NREL—National Renewable Energy Laboratory Extractives in Biomass. https://www.nrel.gov/docs/gen/fy08/42619.pdf.

[67] NREL—National Renewable Energy Laboratory Determination of Sugars, Byproducts, and Degradation Products in Liquid Fraction Process Samples. https://www.nrel.gov/docs/gen/fy08/42623.pdf.

[68] Kulyk K., Palianytsia B., Alexander J.D., Azizova L., Borysenko M., Kartel M., Larsson M., Kulik T. (2017). Kinetics of Valeric Acid Ketonization and Ketenization in Catalytic Pyrolysis on Nanosized SiO_2_, γ-Al_2_O_3_, CeO_2_/SiO_2_, Al_2_O_3_/SiO_2_ and TiO_2_/SiO_2_. Chem. Phys. Chem..

[69] Kulyk K., Borysenko M., Kulik T., Mikhalovska L., Alexander J.D., Palianytsia B. (2015). Chemisorption and thermally induced transformations of polydimethylsiloxane on the surface of nanoscale silica and ceria/silica. Polym. Degrad. Stab..

[70] Kulik T., Palianytsia B., Larsson M. (2020). Catalytic pyrolysis of aliphatic carboxylic acids into symmetric ketones over ceria-based catalysts: Kinetics, isotope effect and mechanism. Catalysts.

