# Supplementary material for: Apricot Seed Shells and Walnut Shells as Unconventional Sugars and Lignin Sources
Source: Molecules. 2023 Feb 2;28(3):1455. doi: 10.3390/molecules28031455 (PMC9918925; doi:10.3390/molecules28031455)
Supplement: Supplementary file 1 [file molecules-28-01455-s001.zip › molecules-2139739-supplementary.pdf]

Apricot seed shells and walnut shells as unconventional sugars and lignin sources

Vita Halysh [1,2], Juan Miguel Romero-García [3,4*], Alfonso M. Vidal [3], Tetiana Kulik [2], Borys Palianytsia [2], Minerva García [5], and Eulogio Castro [3,4]

1 Department of Ecology and Technology of Plant Polymers, Faculty of Chemical Engineering, Igor Sikorsky Kyiv Polytechnic Institute, Peremogy Avenu 37/4, 03056 Kyiv, Ukraine
2 Laboratory of kinetics and mechanisms of chemical reactions on the surface of solids, Chuiko Institute of Surface Chemistry, National Academy of Sciences of Ukraine, General Naumov Str, 17, 03164 Kyiv, Ukraine
3 Department of Chemical, Environmental and Materials Engineering, Universidad de Jaén, Campus Las Lagunillas s/n, Jaén 23071, Spain
4 Center for Advanced Studies in Earth Sciences, Energy and Environment (CEACTEMA), Universidad de Jaén. Campus Las Lagunillas s/n, Jaén 23071, Spain
5 Tecnológico Nacional de México / Instituto Tecnológico de Zitácuaro, Av. Tecnológico No. 186 Manzanillos, C.P. 61534 H. Zitácuaro, Mich., México
* Correspondence: jrgarcia@ujaen.es

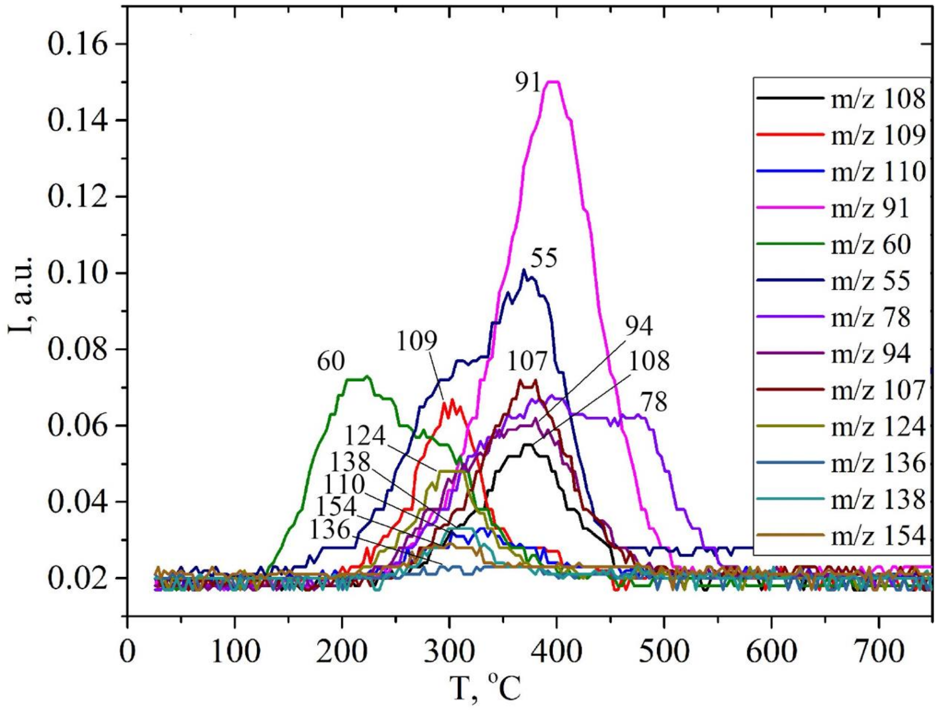

Figure S1. TPD curves for ions with *m/z* 154, 138, 136, 124, 110, 109, 108, 107, 94, 91, 78, 60, 55 obtained via pyrolysis of the solid based on apricot seed shells after alkaline pretreatment

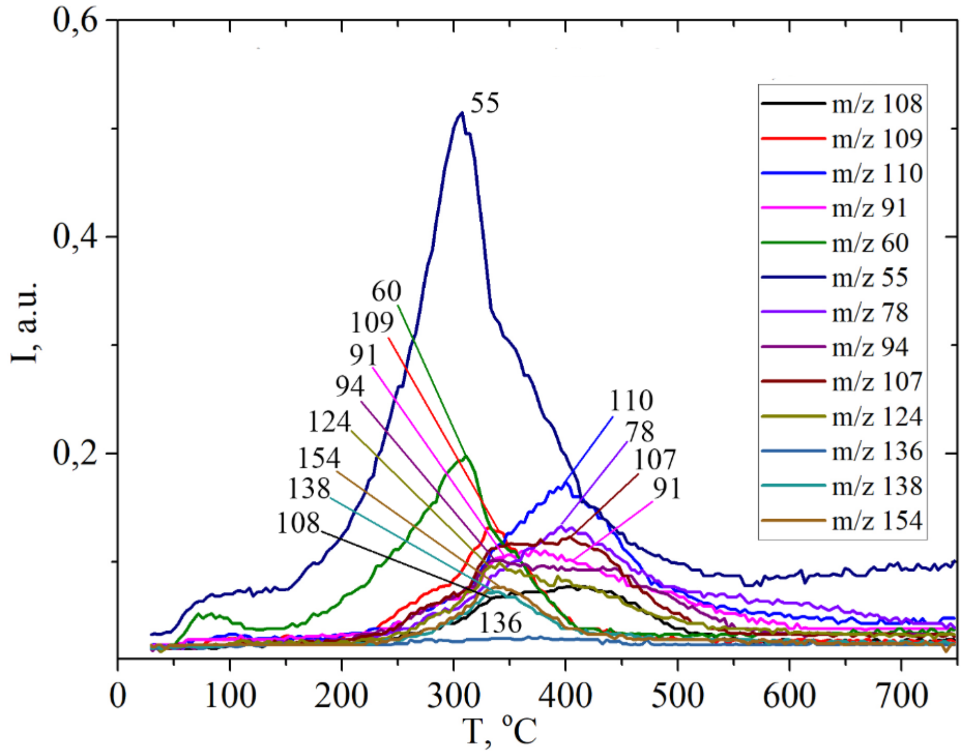

Figure S2. TPD curves for ions with *m/z* 154, 138, 136, 124, 110, 109, 108, 107, 94, 91, 78, 60, 55 obtained via pyrolysis of the solid based on apricot seed shells after acid pretreatment

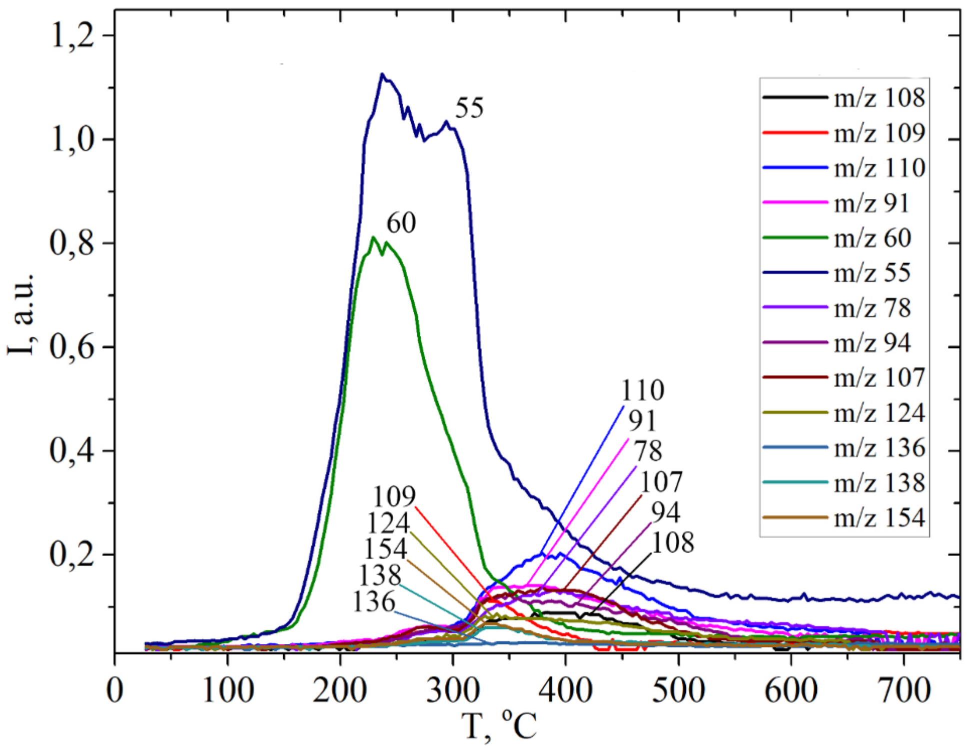

Figure S3. TPD curves for ions with *m/z* 154, 138, 136, 124, 110, 109, 108, 107, 94, 91, 78, 60, 55 obtained via pyrolysis of the solid based on apricot seed shells after pretreatment with steam explosion

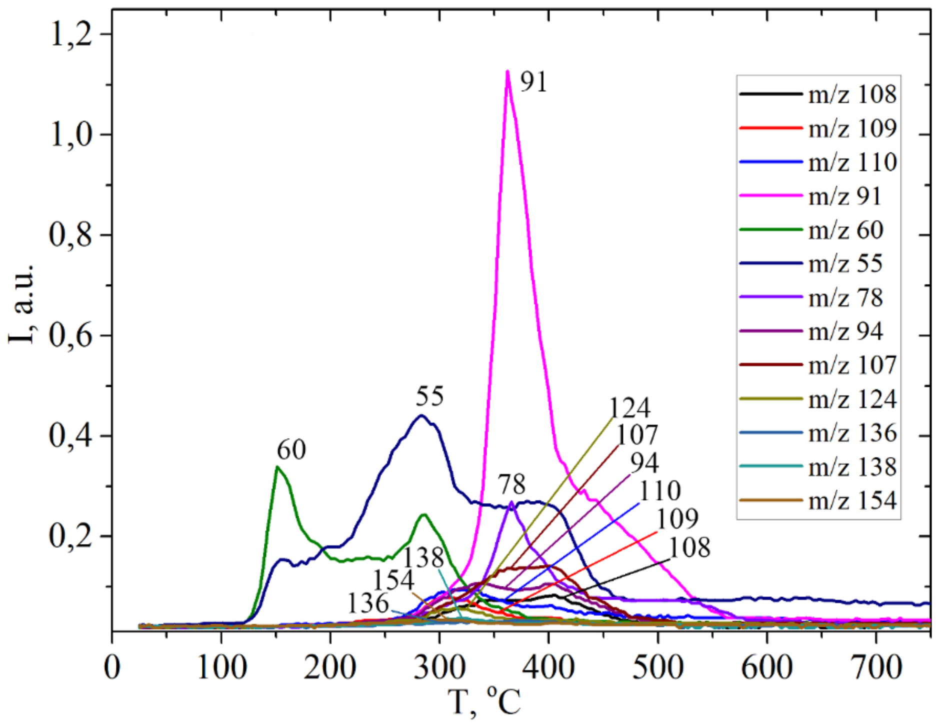

Figure S4. TPD curves for ions with *m/z* 154, 138, 136, 124, 110, 109, 108, 107, 94, 91, 78, 60, 55 obtained via pyrolysis of the solid based on walnut shells after alkaline pretreatment.

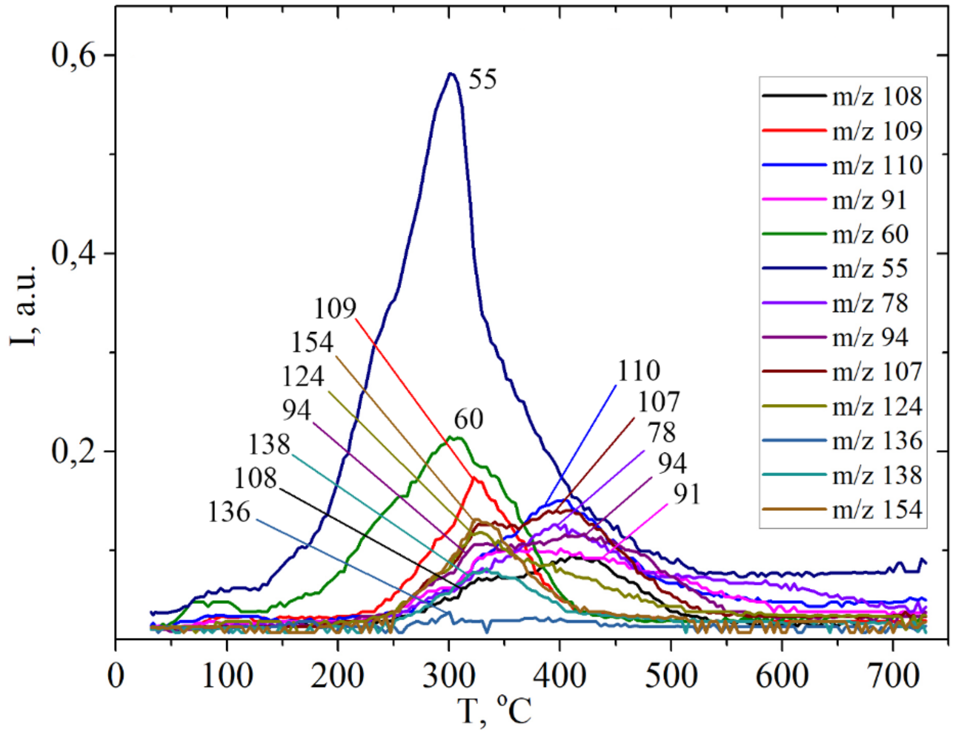

Figure S5. TPD curves for ions with *m/z* 154, 138, 136, 124, 110, 109, 108, 107, 94, 91, 78, 60, 55 obtained via pyrolysis of the solid based on walnut shells after acid pretreatment

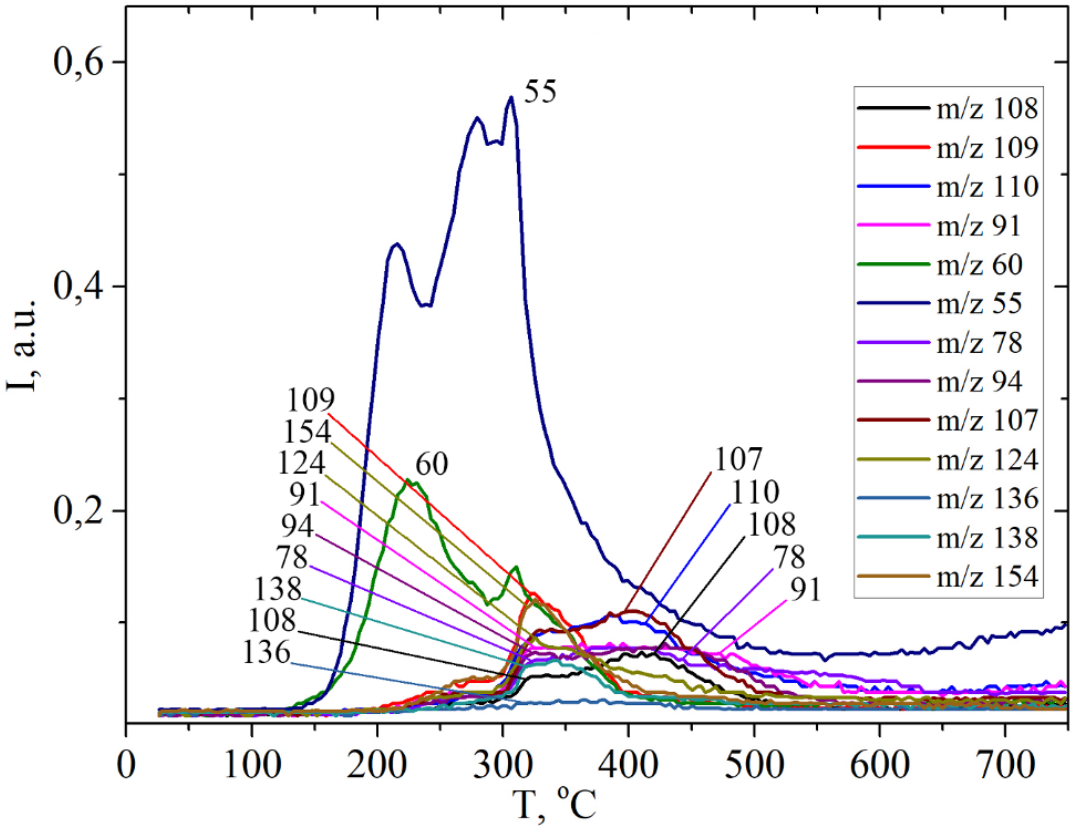

Figure S6. TPD curves for ions with *m/z* 154, 138, 136, 124, 110, 109, 108, 107, 94, 91, 78, 60, 55 obtained via pyrolysis of the solid based on walnut shells after pretreatment with steam explosion
